# A Case of Partial Cystectomy for Spontaneous Intraperitoneal Rupture of the Bladder Revealed from Hemorrhagic and Septic Shock

**DOI:** 10.14789/ejmj.JMJ24-0048-CR

**Published:** 2025-04-10

**Authors:** HIDEYUKI ISOBE, SOU NAKAMURA, NAOKO TAKAZAWA, HANNA SUETSUGU, KAZUNORI KAJINO, SHUU HIRAI, KATSUHITO YUZAWA, SHIGEO HORIE

**Affiliations:** 1 Department of Urology, Juntendo Tokyo Koto Geriatric Medical Center, Tokyo, Japan; 1 Department of Urology, Juntendo Tokyo Koto Geriatric Medical Center, Tokyo, Japan; 2 Department of Pathology, Juntendo Tokyo Koto Geriatric Medical Center, Tokyo, Japan; 2 Department of Pathology, Juntendo Tokyo Koto Geriatric Medical Center, Tokyo, Japan; 3 Department of Urology, Juntendo University, Graduate School of Medicine, Tokyo, Japan; 3 Department of Urology, Juntendo University, Graduate School of Medicine, Tokyo, Japan

**Keywords:** bladder spontaneous rupture, hematuria, partial cystectomy

## Abstract

A 81 - year - old man was transported to the emergency department of nearby hospital due to macroscopic hematuria, abdominal pain, and difficulty moving. When he arrived at the hospital, his blood pressure was decreased and he was in critical condition due to shock. Computed tomography (CT) showed that the bladder was filled with blood clots and ascites retention was recognized. Hemorrhagic and septic shock due to bladder hemorrhage was suspected. After admission, his blood pressure was stabilized after blood transfusion, fluid replacement and antibiotic treatment. But his hemoglobin level did not improved sufficiently on blood sampling. Bladder hemorrhage was considered to be prolonged. A cystography showed a rupture point at the apex of the bladder, and it was determined that surgical treatment was necessary. On the third day of admission, a partial resection of the perforated bladder wall was performed. Postoperatively, hematuria was improved, and the patient was well recovered with no progression of anemia. Spontaneous bladder rupture is a rare disease, and its accurate diagnosis is difficult to make because the clinical symptoms vary. It is important to perform cystourethrography promptly to make a correct diagnosis and to proceed to surgical treatment at the appropriate time.

## Introduction

Spontaneous bladder rupture is relatively rare, although traumatic rupture of the bladder is considered to be a relatively common cause of bladder rupture. Since the treatment varies depending on the circumstances leading to the rupture, the diagnosis must be done quickly and accurately. We report of a case of spontaneous rupture of the bladder into the peritoneal space discovered from hemorrhagic and septic shock due to hematuria, which was treated by the partial cystectomy.

## Case report

**Figure 1:**
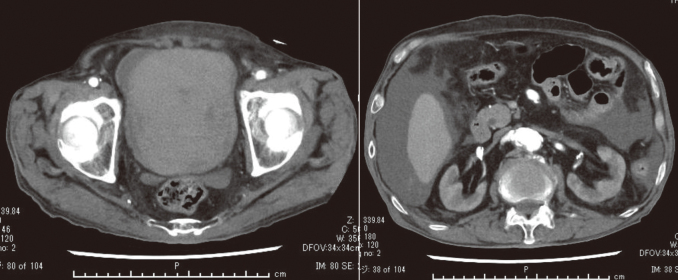
CT shows that the bladder was filled with hematoma on the left and the ascites was detected in the abdominal cavity on the right.

A 81- year - old man was aware of macroscopic hematuria from December 3, 2023 but he ignored the symptoms. He was taking direct oral anticoagulant for atrial fibrillation. He had no history of trauma, surgery, and no other medical history of note. He was transported to the emergency department of a nearby hospital due to abdominal pain and difficulty of moving on December 19, 2023. His condition was that the level of his consciousness was Japan Coma Scale (JCS) 1, the blood pressure was 90/60 mmHg, the heart rate was 100/min., the respiratory rate was 25 times/min., the saturation of percutaneous oxygen was 100% (room air). In blood sampling, the white blood cell count was 7,900/μL, the hemoglobin level was 6.5 g/dL (lower limit of normal 13.4), the hematocrit level was 19.9% (lower limit of normal 40.4), the platelet count was 250,000/μL, the blood urea nitrogen (BUN) level was 69.2 mg/dL (upper limit of normal 21.0), the creatinine level was 5.89 mg/dL (upper limit of normal 1.0), Na 141 mmol/L, K 6.4 mmol/L (upper limit of normal 5.0), Cl 106 mmol/L, the c reactive protein was 15.97 mg/dL (upper limit of normal 0.3), and the procalcitonin level was 80.44 ng/mL (upper limit of normal 0.05). The patient was transfused with red cell concentrate (RBC) 2 Units and fresh frozen plasma (FFP) 2 Units for the diagnosis of severe anemia, acute renal failure, septicemia, and hemorrhagic shock. CT revealed that no obvious neoplastic lesions were observed but the bladder was filled with hematoma, and ascites was detected in the abdominal cavity (Figure 1). The bladder was thought to be the source of the bleeding. No hydronephrosis was observed in either of the bilateral kidneys on CT. The patient referred to the department of general medicine of our medical center for emergency admission on the same day because the inpatient beds were full at the previous hospital.

At the time of admission, His condition was that the level of his consciousness was JCS 1 and his blood pressure was improved to 128/70 mmHg with the rehydration and blood transfusion by the previous physician. The heart rate was 104/min., the respiratory rate was 18 times/min., the saturation of percutaneous oxygen was 100% (room air). His palpebral conjunctiva was pale and he had no cyanosis and no edema. His abdomen was soft and flat, but tenderness was noted throughout the abdomen and no rebound tenderness was noted. Electro-cardiogram was atrial fibrillation rhythm and echocardiography revealed that it had no asynergy, and ejection fraction was 57%, and had mild mitral regurgitation, and the diameter of inferior vena cava was 5 mm. The department of general medicine of our medical center consulted our department at the time of admission. A balloon catheter was placed already in the bladder and we attempted to collect the blood clots by bladder lavage under echo-guided guidance, but the blood clots were so large that the lavage was extremely difficult. On the other hand, urine output was maintained at 700- 1300 ml/day. Since there was concern that repeated excessive flushing might promote bleeding and increase the rupture site, we decided to only observe spontaneous urine flow. As for acute renal failure, there was no evidence of hydronephrosis on CT, and bloody ascites was found in the abdominal cavity, which could be explained by urine reabsorption if the leakage was caused by bladder rupture. As the patient’s vital signs were stabilized by supplemental fluids, we decided to proceed with a close examination of the site of bleeding in the bladder and further blood transfusion for surgical treatment, and to consider the timing of elective surgery. Urine cultures have detected Corynebacterium species 107 colony forming units/mL. The patient continued to receive 6.75 mg/day of piperacillin tazobactam (PIPC/TAZ) as an antibiotic. After admission, the indwelling bladder catheter was frequently obstructed and required irrigation, but somehow urine drainage was preserved. Urine cytology at the time of admission was negative, and on December 20, an echo-guided abdominocentesis was performed, which revealed bloody ascites.

**Figure 2:**
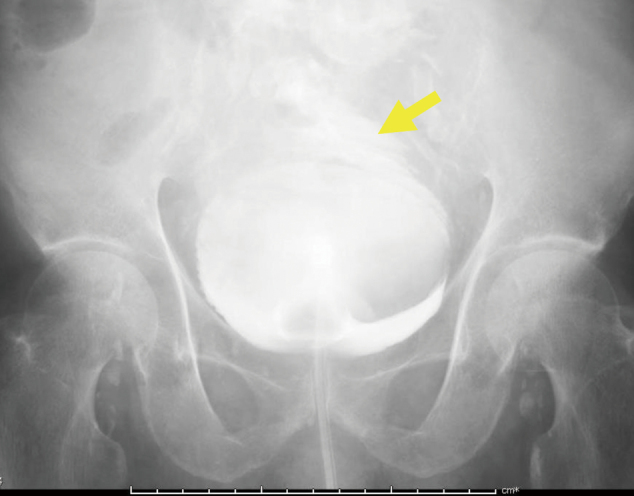
Yellow arrow shows that the contrast medium was confirmed to leak from the apex of the bladder into the abdominal cavity with cystography.

The creatinine level of ascites fluids was 8.62 mg/dL, which was higher than the serum level. Ascites cytology was negative. On December 21, cystography was performed and contrast medium was confirmed to leak from the apex of the bladder into the abdominal cavity (Figure 2). Renal function and K levels on admission showed blood urea nitrogen (BUN) 72 mg/dL, Creatinine 5.27 mg/dL, and K 5.6 mmol/L. The next day data were BUN 74 mg/dL, Creatinine 4.94 mg/dL, and K 5.5 mmol/L. The data on the day of surgery were BUN 54 mg/dL, Creatinine 3.74 mg/dL, and K 4.7 mmol/L. Although BUN and creatinine levels were high, dialysis was not immediately considered because urine output was maintained, CT clearly showed fluid retention in the abdominal cavity and K levels gradually improved.

**Figure 3:**
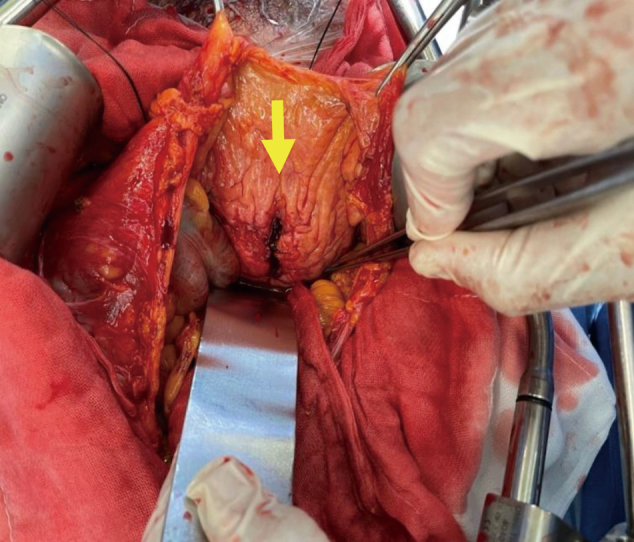
Yellow arrow shows that a 2 cm rupture was observed near the apex of the posterior wall of the bladder communicated with abdominal cavity.

We diagnosed a bladder rupture into the abdominal cavity and decided to perform an open bladder repair. On December 22, laparotomy was performed in the lower abdominal midline incision under general anesthesia. A trans-retroperitoneal high bladder incision was placed. After removing the intravesical hematoma, a 2 cm rupture was observed near the apex of the posterior wall of the bladder with intra-abdominal cavity (Figure 3). There were no bleeding points in the bladder after removal of the blood specimen. There were no obvious mucosal abnormalities, diverticulum, trabeculation, tumors, or stones in the bladder other than the ruptured area. The peritoneum was opened just above the peritoneal reflection. Intra-abdominal observation revealed no obvious abnormal findings other than bloody ascites.

The bladder wall was resected in an oval shape 2 cm away from the injured site. The ruptured site was repaired from the peritoneal side and inside the bladder respectively with interrupted sutures in two layers with 2-0 synthetic absorbable sutures. Drains ware placed in the right subhepatic space of the abdominal cavity and in the left lateral extraperitoneal space of the bladder. The duration of operation was 3 hours and 14 minutes, and the intraoperative blood loss was 2572 g including hematoma.

**Figure 4:**
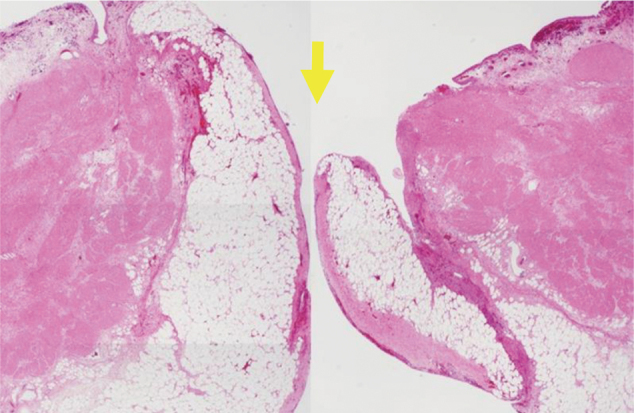
Hematoxylin and eosin stained image (×100) Yellow arrow shows that no obvious malignant findings, chronic inflammation, or thinning of the muscular layer in the bladder wall at the ruptured site was not recognized by the pathological examination.

Pathological examination revealed no obvious malignant findings, chronic inflammation, or thinning of the muscular layer in the bladder wall at the ruptured site (Figure 4). On January 4, 2024, cystography showed no leakage, and the urethral catheter was removed on January 9. He was able to void on his own and had no residual urine. Rehabilitation was started for postoperative disuse syndrome, and the patient was transferred to the hospital for continued rehabilitation on February 9, 2024.

## Discussion

Bladder rupture is classified into traumatic and spontaneous rupture according to etiology, and spontaneous rupture is defined as “any rupture of the bladder into the abdominal or pelvic space occurring without trauma”^1)^. The majority of ruptures are traumatic (96.6%), and spontaneous ruptures are relatively rare^2)^. Spontaneous ruptures are further divided into symptomatic and idiopathic ruptures of unknown cause. Symptomatic ruptures are considered to have a bladder wall cause (e.g., bladder tuberculosis, bladder cancer, bladder stones, cystitis, radiation injury, etc.) and those caused by bladder hyperextension (after alcohol consumption, prostate enlargement, urethral stricture, neurogenic bladder, etc.). Spontaneous bladder rupture after alcohol consumption used to be more common^3)^, but reports of spontaneous bladder rupture due to neurogenic bladder from cervical cancer surgery and radiation injury from radiotherapy are increasing^4)^. Bladder rupture is classified into three types according to the type of rupture: extraperitoneal rupture, intraperitoneal rupture, and a combination of both, with frequencies of 60%, 30%, and 10%, respectively^5)^.

In bladder rupture, we consider extraperitoneal rupture to be the most common type of rupture because of the high frequency of traumatic cases caused by pelvic fractures and the bladder neck injury. However, if blunt external force is applied while the bladder is filled with urine, intraperitoneal rupture is possible as well as spontaneous rupture. In contrast, spontaneous rupture is often intraperitoneal^6)^. In this case, preoperative CT showed marked ascites, and rupture into the peritoneum was suspected. The most common site of rupture is the apex of the bladder. This is thought that the anterior and lateral walls of the bladder are anatomically fixed by the pelvic bones and pelvic muscles, whereas the apex of the bladder is vulnerable because it abuts the abdominal cavity and has the thinnest muscle layer with the least supportive tissue^7)^. In general, bladder rupture is difficult to diagnose by CT scan alone^8)^ and cystography is most useful. Blood test findings are known to show acute renal failure due to reabsorption of urine leaked into the abdominal cavity by the peritoneum and elevated urea nitrogen and creatinine^9)^. The present case presented with similar laboratory findings to this, but it is difficult to differentiate it from renal failure due to peritonitis or shock and requires careful management.

Compared to previous literature reports, this case is atypical in that the patient had macroscopic hematuria a few days before the onset of the disease and a large accumulation of blood clots in the bladder at the time of presentation. He had no history of radiation therapy, and based on this postoperative voiding status, it was assumed that he had no neurogenic bladder, lower urinary tract obstruction, or other urinary disorder prior to the onset of his disease. We speculated that hematuria due to urinary tract infection preceded the bladder rupture, and that the blood clots accumulation caused bladder tamponade, which led to spontaneous rupture due to hyperextension of the bladder when it was distended. However, histopathological examination of the bladder revealed no evidence of chronic inflammation, malignant findings or thinning of the muscle layer, suggesting a fragile bladder wall, and it is unclear why the hyperextension caused a rupture wound that extended beyond the peritoneum into the abdominal cavity.

Some reports have shown that conservative treatment of the bladder rupture, such as urinary drainage with an indwelling bladder catheter and administration of the antibiotics for peritonitis, can be curative^10-12)^.

However, if urinary drainage is difficult or if intestinal perforation cannot be rule out due to complications of free air, urgent laparotomy is necessary.

While conservative cure can be expected only with urinary drainage in the case of extraperitoneal rupture, intraperitoneal rupture is associated with a high risk of deterioration of general condition due to infection, and recurrence is often reported in patients with a history of pelvic radiation therapy for cancer or in elderly patients, so surgical treatment is preferable. Each case is treated differently depending on the circumstances leading to the rupture, so the decision for surgical treatment must be made quickly and accurately. In this case, the patient was in the state of hemorrhagic and septic shock when he came to our hospital. Since the patient was at high risk for surgery to remain in this state but urine could be drained with an indwelling bladder catheter, blood transfusion and ascites examination were performed first, giving priority to conservatively stabilization of the general condition. Surgical treatment could be performed because the patient’s condition was stabilized by blood transfusion and antibiotic treatment, and the rupture site was identified by cystography and was prepared adequately for the laparotomy.

## Conclusion

We experienced a case of partial cystectomy for spontaneous rupture of the bladder into the abdominal cavity, which identified due to hemorrhagic and septic shock due to hematuria.

## Funding

No funding was received.

## Author contributions

NS analyzed and interpreted patient data and made significant contributions to the writing of the manuscript. All authors read and approved the final manuscript.

## Conflicts of interest statement

There is no COI to disclose for this paper.
